# Clinical Applications of 3‐Dimensional Printing Technology in Hip Joint

**DOI:** 10.1111/os.12468

**Published:** 2019-07-18

**Authors:** Run‐zhi Xia, Zan‐jing Zhai, Yong‐yun Chang, Hui‐wu Li

**Affiliations:** ^1^ Shanghai Key Laboratory of Orthopaedic Implants, Department of Orthopaedic Surgery, Shanghai Ninth People's Hospital Shanghai Jiaotong University School of Medicine Shanghai China

**Keywords:** Hip Joint, Patient‐Specific Modeling, Printing, Three‐Dimensional, Prosthesis Design

## Abstract

Three‐dimensional (3D) printing is a digital rapid prototyping technology based on a discrete and heap‐forming principle. We identified 53 articles from PubMed by searching “Hip” and “Printing, Three‐Dimensional”; 52 of the articles were published from 2015 onwards and were, therefore, initially considered and discussed. Clinical application of the 3D printing technique in the hip joint mainly includes three aspects: a 3D‐printed bony 1:1 scale model, a custom prosthesis, and patient‐specific instruments (PSI). Compared with 2‐dimensional image, the shape of bone can be obtained more directly from a 1:1 scale model, which may be beneficial for preoperative evaluation and surgical planning. Custom prostheses can be devised on the basis of radiological images, to not only eliminate the fissure between the prosthesis and the patient's bone but also potentially resulting in the 3D‐printed prosthesis functioning better. As an alternative support to intraoperative computer navigation, PSI can anchor to a specially appointed position on the patient's bone to make accurate bone cuts during surgery following a precise design preoperatively. The 3D printing technique could improve the surgeon's efficiency in the operating room, shorten operative times, and reduce exposure to radiation. Well known for its customization, 3D printing technology presents new potential for treating complex hip joint disease.

## Introduction

Three‐dimensional (3D) printing, or rapid prototyping, is a tool used to apply a group of techniques to quickly fabricate a scale model of a physical object using 3D computer‐aided design (CAD) data. The origin of 3D printing can be traced back to the 1960s when Professor Herbert Voelcker described theories and algorithms for 3D model fabrication^1^. Carl Deckard developed a technique to bind metal powers to create a 3D model in the University of Texas in 1987 and Charles Hull patented the first 3D printer in California in 1988^2^. 3D printing has been used in the medical industry since the early 2000s, initially in the production of patient‐specific prostheses and dental implants^3^. Medical applications of 3D printing techniques in orthopaedic departments mainly include 3D printing of a bone model, custom prostheses, and patient‐specific instruments (PSI). By introducing the application of 3D printing in different cases of hip joint diseases, this review may provide some novel ideas for orthopaedic surgeons to deal with complex issues.

### *Procedure of 3‐Dimensional Printing*

Images of patients’ hip joints should be obtained through CT scan and saved in Digital Imaging and Communications in Medicine (DICOM) format and imported into Materialise Interactive Medical Image Control System (MIMICS) or 3D Slicer, or some other medical imaging processing software^4^. Different kinds of 3D processing software are shown in Table 1.

**Table 1:** Three‐dimensional software

Software	Description
MIMICS^5^	Import DICOM, JPEG, TIFF, BMP, X‐ray or raw image data and export 3D models for 3D analysis, finite element analysis meshing, design or 3D printing; MIMICS could perform dedicated anatomical analysis, create accurate virtual 3D models and plan a surgical procedure virtually; MIMICS was the most widely used software (22 in 33 articles mentioned the 3D printing software)
3D Slicer^4^	Import DICOM images and a variety of other formats; 3D Slicer could carry out analysis and visualization of diffusion tensor imaging data and automatic image segmentation
Blender^6^	DICOM image processing software which supports the entirety of the 3D pipeline: modeling, rigging, animation, simulation, rendering, and compositing
OsiriX^7^	A DICOM viewer and a multiplane reconstruction tool
Geomagic Studio^8^	Import .STL file to deliver precise digital 3D models and computer‐aided design assemblies of physical objects for use in designing, engineering, and manufacturing
MeshLab^9^	Import .STL file to provide a set of tools for editing, cleaning, healing, inspecting, rendering, texturing, and converting meshes; offers features for processing raw data produced by 3D digitization devices and for preparing models for 3D printing
Solideworks^10^	Import .STL file to utilize a parametric feature‐based approach to create models and assemblies
Meshmixer^11^	Import .STL file to carry out 3D sculpting, surface stamping, hole filling, bridging, boundary zippering, and auto‐repair; enable plane cuts, mirroring, remeshing, mesh simplification, mesh mixing, and mesh smoothing
NetFabb^4^	Import .STL file to repair and edit models and realize design optimization
MakerBot^12^	Import .STL file to optimize and streamline the design files and fine‐tune their settings for optimum results when printing
Materialise 3‐Matic^13^	Import .STL file to perform design optimization and modification on mesh level; allow creation of directly printable internal and external structures that add extra strength, provide cushioning, increase porosity, or simply reduce the weight of the design
Unigraphics NX^14^	Import .STL file or not to design a model and obtain rapid manufacturing finished by using included machining modules
Pro/Engineer^15^	Import .STL file or not to provide solid modeling, assembly modeling, 2D orthographic views, finite element analysis, and direct and parametric modeling
Simplify3D software^11^	An .STL editor software that is compatible with more 3D printers than any other software available
Materialise Magics^16^	An .STL editor software which specializes in geometrically correct fixing and repairing textures and colors

BMP, Bitmap; DICOM, Digital Imaging and Communications in Medicine; JPEG, Joint Photographic Experts Group; STL, stereolithographic; TIFF, Tag Image File Format.

**Figure 1:**
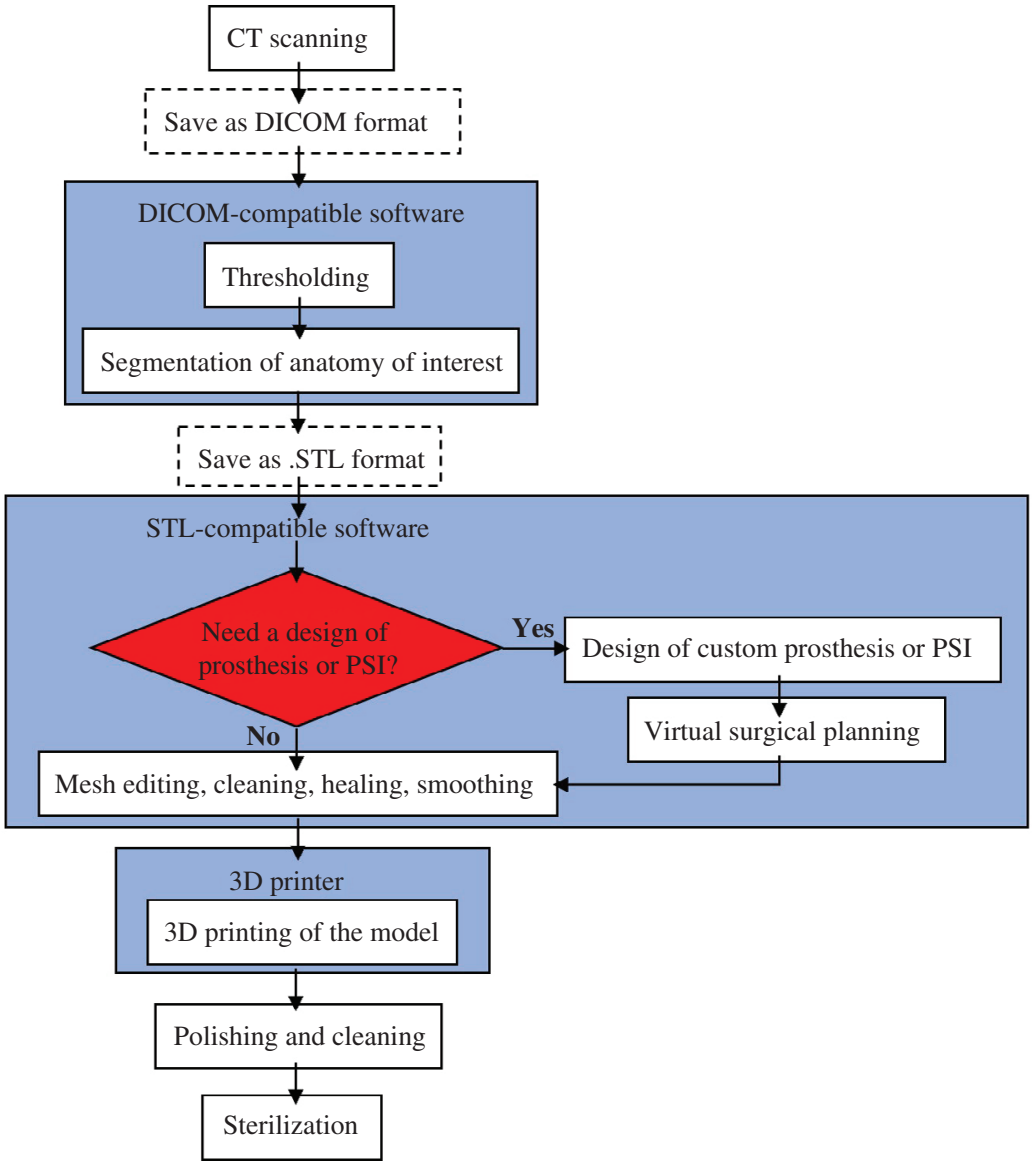
Whole process of three‐dimensional (3D) printing. CT scan obtained from the patients should be saved as digital imaging and communications in medicine (DICOM) format and imported into DICOM‐compatible software to conduct thresholding and segmentation of anatomy of interest. Then, the file should be saved in stereolithographic (.STL) format to be communicated to the STL‐compatible postprocessing software for further mesh editing, cleaning, healing, and smoothing. We can also design custom prostheses or patient‐specific instruments (PSI) for virtual surgical planning if necessary. Finally, the. STL file is imported into a 3D printer to result in the formation of 3D structures.

Image thresholding is performed, which allow bones to be differentiated from the surrounding soft tissue based on bone and soft tissue densities on the CT scan. A radiologistsegments the bones in regions where partial volumes fall below the threshold. Subsequently, a 3D image of the isolated anatomy of interest will be created using MIMICS or 3D slicer^17^. Accurate segmentation of the bones is key for reproduction of relevant anatomy in the 3D model. It requires technical skill and for the radiologist to be experienced with the software segmentation tools. The custom cage and PSI can be designed using MIMICS software^18^. After segmentation of the relevant anatomy, the file should be saved in stereolithographic (.STL) format to be communicated to the postprocessing software and 3D printer^19^. The .STL file can be imported into Netfabb, MeshLab, or Meshmixer so that the model can be further remeshed, stitched, and surface wrapped. Finally, the .STL file will be imported into a 3D printer, which uses metal or plastic powder and other special materials with a laser beam or hot melt nozzle to print in the 2‐dimensional (2D) *x*–*y* plane bonded into a cross‐sectional shape, and then in the *z*‐coordinate layer stack, to ultimately result in the formation of 3D structures^20^. The whole 3D printing process is shown in Fig. 1. Three‐dimensional techniques and materials that have been mentioned in relevant articles are demonstrated in Table 2.

**Table 2:** Three‐dimensional techniques and materials

3D Printing techniques	Description	Materials
Stereolithography (SLA)^21^	A laser selectively illuminates the transparent bottom of a tank filled with a liquid photo‐polymerizing resin, and the laser can polymerize the resin in layers as the tank descends deeper and deeper	Photopolymers
Fused deposition modeling (FDM), also referred to as free form fabrication (FFF)^22^	A filament of plastic material is fed through a heated moving head that melt, extrude and deposit the material layer after layer in the desired shape; a moving platform lowers after each layer is deposited; additional vertical support structures are needed to sustain overhanging parts for this kind of 3D printing technology	Thermoplastics (such as acrylonitrile butadiene styrene, polylactic acid, polycarbonate, polyamide, and polystyrene)
Selective laser sintering (SLS)^17^	Uses a laser as the power source to sinter powdered material; in contrast with SLA and FDM, which most often require special support structures to fabricate overhanging designs, SLS does not need a separate feeder for support material because the part being constructed is surrounded by unsintered powder at all times	Plastic, metal, ceramic, or glass powders
Selective laser melting (SLM) or direct metal laser sintering (DMLS)^21^	Uses a high power‐density laser to melt and fuse metallic powders together; SLM is considered to be a subcategory of selective laser sintering (SLS)	Metal powders (such as titanium)
Electron beam melting (EBM)^23^	The raw material (metal powder or wire) is placed under a vacuum and fused together from heating by an electron beam; this technique is distinct from SLS as the raw material fuses having completely melted	Metal powders
Multi‐jet modeling^21^	The powder bed is heated uniformly at the outset; a fusing agent is jetted where particles need to be selectively molten, and a detailing agent is jetted around the contours to improve part resolution; while lamps pass over the surface of the powder bed, the jetted material captures the heat and helps distribute it evenly	Plastics
PolyJet printing^21^	PolyJet works by jetting photopolymer materials in ultra‐thin layers onto a build platform; each photopolymer layer is cured by UV light immediately after it is jetted, producing fully cured models that can be handled and used immediately, without post‐curing; the gel‐like support material, designed to support complicated geometries, is subsequently removed by water jetting	Photopolymers

## Three‐Dimensional Printed Bony Model

Appreciation of the abnormality in the hip joint may not always be obtained on a 2D screen. A 3D‐printed model provides visual and tactile sensation of the impaired pelvic anatomy, which brings an improved understanding of the anatomy to surgeons and facilitates preoperative planning^24^.

### *Acetabular Fracture*

The rate of accidental acetabular fracture has been increasing in recent 5 years^25^. In view of the seriousness of acetabular fractures, they should be treated after adequate preparation and planning. In addition, the surgical effect for acetabular fractures positively correlates with the extent of bone reduction. At Nanfang Hospital on 24 January 2014, in a world first, surgery for an acetabular fracture was conducted using 3D printing^26^. Yu *et al*. 3D‐printed the pelvic model of a patient with a complex acetabular fracture to choose the best approach and to decide on the preoperative plan, thereby shortening the operative time^27^. Liu *et al*. printed the affected semi‐pelvic and femur model of a patient with a complex acetabular fracture. Through preoperative surgery with the 3D printing tool, they determined the directions and the lengths of screws and recorded the position of plates. As a result, radiological views showed that the acetabulum was anatomically reduced and the hip joint was congruous^28^. Several similar studies have been reported in recent years^5^, ^6^, ^12^, ^29^, ^30^, ^31^, ^32^.

Mirror images have already been used for orthopaedic surgery such as osteotomy. Except for some rotational parameters, few studies have shown the symmetry of both hemipelvis in healthy patients. Chana‐Rodríguez *et al*. reported a case of a patient with the diagnosis of complex acetabular fracture; they used a specular healthy hemipelvis model (reverse engineering not the affected hemipelvis) for preoperative planning due to the advantage of reproducing the injured area without fractures of bone fragments. The fracture was represented directly on the model with indelible ink. Plate pre‐contouring was implemented, adapting their shape to the anatomical contours without further corrections to the patient during surgery. The postoperative CT scan showed an anatomical reduction and no slits were observed between the plates and the acetabular surface^9^. Another two studies also created mirror pelvic models of the patients to conduct preoperative planning^7^, ^33^. However, because almost all the cases lack direct comparison between preoperative simulation and the actual surgical procedure, it is hard to show the efficacy of preoperative simulation objectively. Therefore, Liu *et al*. contrasted postoperative CT scanning with preoperative simulation images. He found that although some simulative screws are longer than the actual screws, most screws and plates could overlap the simulation images^34^.

### *Femoral Intertrochanteric Fracture*

Proximal femoral nail anti‐rotation (PFNA) is an intramedullary gear that includes a helical blade inserted by impaction; the resulting bone compaction around the blade helps to prevent rotation and varus collapse^35^. Previous studies have reported low complication rates and satisfactory results following the use of PFNA^6^; thus, it has become the most commonly used method of treating unstable femoral intertrochanteric fracture (ITF)^36^. Zheng *et al*. conducted a case‐control study of femoral intertrochanteric fractures, with patients divided into two groups: 19 patients underwent PFNA with 3D printing‐rapid prototyping (3DP‐RP), whereas the other 20 patients underwent conventional PFNA treatment. Simulated fracture reduction was conducted on the model. Eventually, it was demonstrated that the 3DP‐RP assisted procedure resulted in more effective reduction of the femoral neck‐shaft angle (NSA). Furthermore, patients undergoing 3DP‐RP experienced a significant reduction in duration of surgery (*P* < 0.01), as well as reductions in intraoperative (*P* = 0.02) and postoperative (*P* = 0.03) blood loss compared with conventional surgery. The postoperative follow‐up indicated that patients in the 3DP‐RP group had a shorter time to ambulation compared with patients that underwent the conventional procedure. The results suggest that the 3DP‐RP technique can create a proper model of the ITF, which facilitates surgical planning and fracture reduction, thus improving the efficiency of PFNA surgery for ITF^37^.

### *Total Hip Arthroplasty*

**Figure 2:**
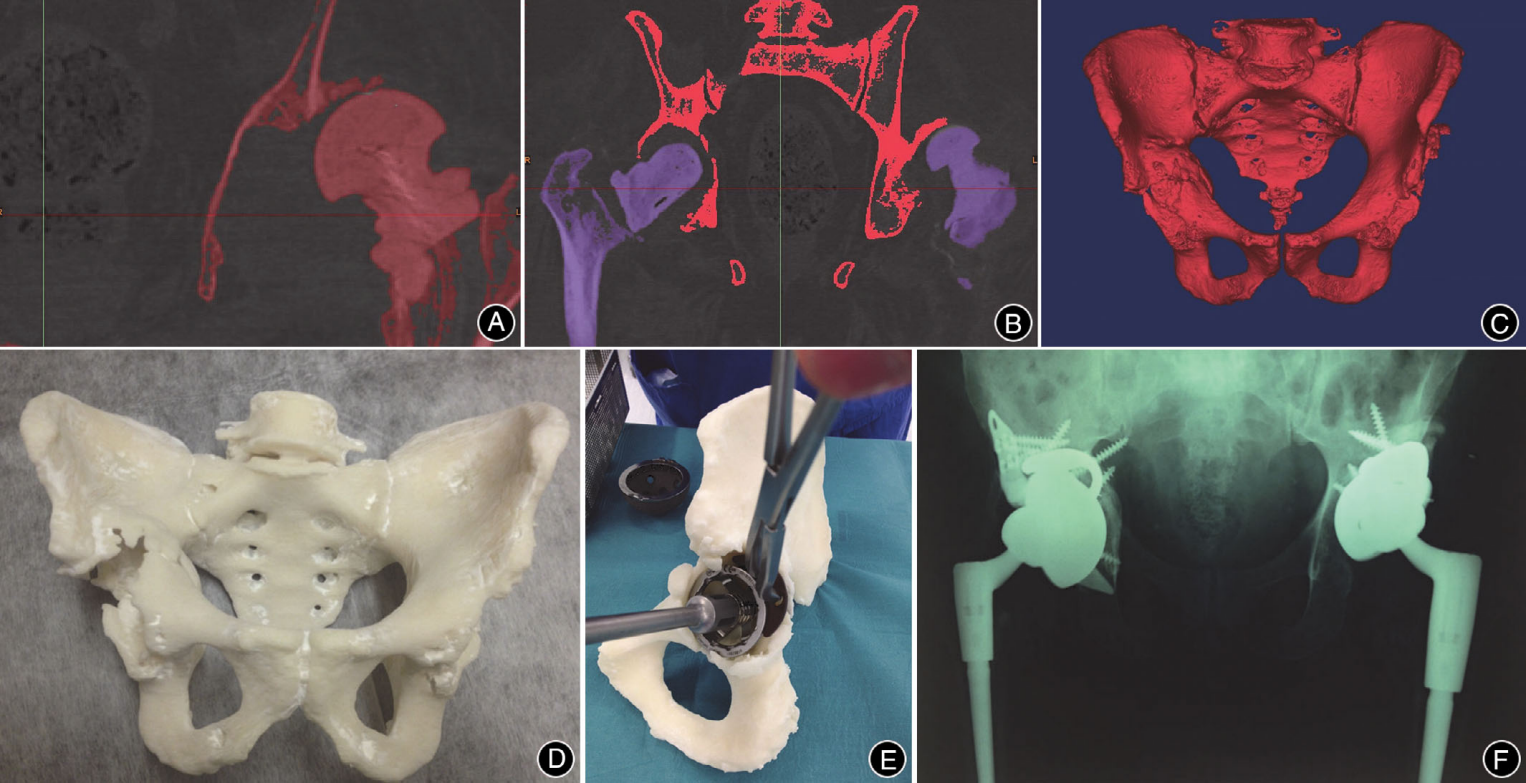
A, Image thresholding was performed by using software, which allowed for bone to be differentiated from surrounding soft tissue based on bone and soft tissue densities on the CT scan; B, Using the region growing process, both femurs were digitally segmented from their corresponding pelvis. The red pelvis will be retained, while the purple femurs will be removed; C, Once both femurs were erased, the 3D isolated image of pelvis (namely, the anatomy of interest) was created; D, The final life‐size 3D‐printed pelvis model, providing the surgeon with visual and tactile appreciation of the defects; E, Acetabular cup, augment, and buttress sizes, as well as cage dimensions were selected and trialed in preoperative surgical stimulation using a 3D‐printed pelvis; F, Postoperative anteroposterior pelvic plain film radiographs showed satisfactory revision total hip arthroplasty *in situ* 17.

Because of the distorted anatomy of the acetabulum and the proximal femur, adult patients with developmental dysplasia of the hip (DDH) have leg length discrepancy and will develop secondary osteoarthritis; therefore, total hip arthroplasty (THA) in these patients represents a valuable procedure^38^. Placement of the cup for patients with DDH is technically difficult because normal anatomic landmarks are vague. Care needs to be taken in adjusting the cup size, the inclination, the cup anteversion, and the coverage. Zerr *et al*. present a case report of 3D printing technology in a THA revision. They created a full‐scale model of the affected pelvis and femur for trialing of the acetabular component to determine the cup size, the position, the screw placement, and the need for reaming. By preoperative simulation, postoperative radiation showed that the prosthesis was stable with multiple screw fixation^4^. Similarly, Hughes *et al*. made a hemipelvis model and a full pelvic model to conduct complex revision hip arthroplasty on two patients. Life‐size models allow precise surgical simulation, enabling preoperative cup, augment, and buttress sizing, as well as cage templating and screw trajectory optimization, allowing for improved accuracy and, thus, reducing the chance of intraoperative neurovascular injury. As a result, complicated revision cases can be carefully evaluated and classified preoperatively, giving the surgeon an opportunity to treat the patient with improved surgical precision (Fig. 2)^17^. Another two studies also used 3D‐printed models to undertake preoperative planning before operations for DDH: one by Xu *et al*., which includes 14 cases^39^, and another by Bagaria, which includes 50 cases.

### *Periacetabular Osteotomy*

The Bernese periacetabular osteotomy (PAO) developed by Ganz *et al*. is an effective joint preserving procedure for young adults with DDH^40^. The main difficulty of PAO is surgically determining the direction and degree of rotation of the osteotomized segment. Zhou *et al*. conducted a cadaveric study to explore the efficacy of a 3D‐printed navigation template for PAO. All participating surgeons found that the 3D‐printed bone‐drilling template and angle fix wedge greatly facilitated intraoperative rotation and fixation, and allowed for accurate final placement of the acetabulum based on the preplanned data^8^. However, anterior impingement after PAO, which occurs at a high rate (47.8%) in male patients^41^, can lead to unsatisfactory clinical results and progression of osteoarthritis. Although predicting anterior impingement before PAO surgery is difficult, the optimal position of the osteotomized fragment can be decided preoperatively to avoid anterior impingement. Fukushima *et al*. designed the osteotomy line according to the measurement and decided on the position of the osteotomized fragment on the 3D pelvic model preoperatively. Their method can be used to simulate motion preoperatively, to analyze the impingement between the osteotomized fragment and the femoral bone, ultimately avoiding anterior impingement after PAO. As a result, they finished the PAO successfully and prevented anterior impingement after the operation^42^. For femoroacetabular impingement per se, Wong *et al*. showed that 3D models of the patient can be used in preoperative planning to determine the extent and location of osteoplasty for femoroacetabular impingement surgery^11^.

### *Slipped Capital Femoral Epiphysis*

With an annual incidence of 10.8 cases per 100 000 children, slipped capital femoral epiphysis (SCFE) is the most common hip disorder in adolescents aged 9 to 16 years^43^. Three‐plane proximal femoral osteotomy (TPFO) has been described to correct the extension, varus, and external rotation deformities characteristic of SCFE, with low rates of femoral head avascular necrosis^44^. Fifteen children treated with TPFO due to SCFE were included in Cherkasskiy's study. Ten patients were treated by a single surgeon with or without a 3D model for preoperative planning, and compared with five patients treated by two senior partners without the use of a model to evaluate for a learning curve^45^. On average, surgical time decreased by 45 min and 38 min, and fluoroscopy time decreased by 50% and 25% in the model group compared with the no‐model and two senior groups, respectively. Although statistically insignificant, these reductions in surgical time and fluoroscopy time may be considered clinically significant.

## Custom Prosthesis

Prosthetic reconstruction is a promising treatment because of immediate stability, and the possibility of rapid recovery as well as the ability to perform early weight‐bearing exercises^46^. In the past, patient‐specific designed prostheses with complex shapes have been difficult to produce because of the limitations of the traditional manufacturing technology. More importantly, long‐term non‐integration between traditional implant and host bone may result in inevitable reconstruction failure^47^. 3D printing technology, also called rapid prototyping, may provide a solution. It can be used to fabricate anatomy‐conforming prostheses of any shape with a porous metal surface allowing osseointegration at the bone–implant junctions^48^.

### *Fracture*

The quadrilateral area is often involved in complex acetabular fractures. Because of the special anatomy, fractures of the quadrilateral area are hard to expose and conducting internal fixation *via* the ilioinguinal approach is difficult. There used to be no ideal internal fixation object; therefore, fractures of the quadrilateral area were formerly difficult to treat. Nowadays, 3D printers have enabled rapid structuring of versatile shapes with minimal infrastructure by simply converting custom designed shapes into physical objects. Designed with the assistance of 3D printing, Nanfang Hospital printed the pelvic model of 8 patients with complex acetabular fractures and designed a novel plate in accordance with the specular healthy hemipelvis model^10^. The novel plate which looks like a wing has three points to be fixed on the pelvis: the posterior point is fixed above sciatic notch, the anterior point on the pubic branch near pubic symphysis, and the inferior point on the sciatic spine. As a result, all eight patients showed primary healing, without hematoma, or vascular and nerve damage. The postoperative CT scan showed a favorable anatomical reduction^10^.

### *Hip Deformity*

Total hip arthroplasty has been used in China since the late 1980s. Successful replacements in cases of severe hip disease have brought relief to many patients. THA not only reduces the pain of patients but also allows them to regain their physical ability. After decades of clinical application, THA has become a standard treatment for hip disease. Hip deformity surgery programs are challenging: they need to optimize prosthetic model choice, accuracy of the prosthesis placement, and the degree of deformity correction for each patient^49^. The conventional THA is not sufficiently individualized for all cases, and this leads to frequent deviations of the implanted prostheses. Wang *et al*. designed a study to compare the clinical data between the use of the 3D printing technique and conventional hip replacement in THA for severe hip deformities. They made 3D‐printed pelvic models and acetabulum prostheses for the 3D group^20^. In their study, no significant differences were found between two sides of a hip in terms of anterior and lateral femoral anteversion, neck‐shaft, acetabular or sharp angles in the patients of the 3D printing group. In contrast, the average anteversion angles of the ipsilateral and contralateral hip sides in patients of the conventional hip replacement group were significantly different, which indicated that the 3D prostheses were closer to the anatomies of patients. In addition, the time to weight loading in the 3D printing group was less than that for the conventional hip replacement group and the postoperative Harris scores were higher in the 3D printing group, indicating that the 3D‐printed prostheses allow for better coordination to human biomechanics^20^. A study by another group showed results like these on surgeries performed on 22 DDH cases^50^.

**Figure 3:**
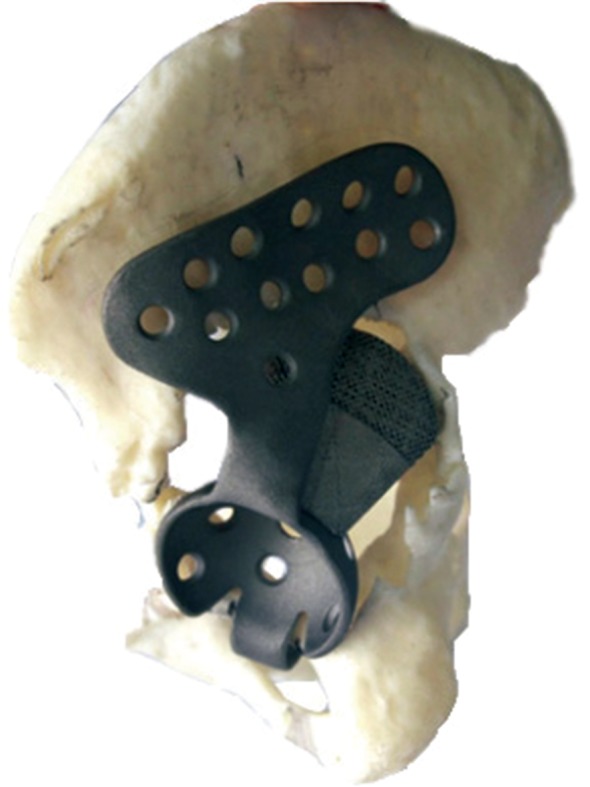
A custom cage with an iliac braid to ensure enough screws could be used for firm fixation and a 3D‐printed augment to the superior surface of the cage for stable support^51^.

Revision hip arthroplasty is conducted when a primary THA fails due to a variety of reasons, such as aseptic loosening (50%), instability (16%), infection (15%), debilitating pain, periprosthetic fractures, or component failure^1^. Applying acetabular revision for people with massive bone defects remains one of the most difficult challenges in hip arthroplasty for surgeons. Many methods have been developed, such as revision with structural grafts, oblong cups, reinforcement rings, a cementless modular revision system, and use of a jumbo acetabular component. Because the remaining acetabular rim cannot maintain adequate initial component stability, there are few acceptable options for severe acetabular bone defects. Li *et al*. used individualized custom cages in a group of 26 patients with severe (Paprosky IIIB) bone defects in revision THA (Fig. 3). He showed that the individualized custom cages result in improved Harris hip scores, restoration of a closer‐to‐normal hip center, and low incidence of surgical complications^51^. Mao *et al*. used custom cages in revision hip arthroplasty for 23 patients with massive acetabular bone defects of Paprosky type III. Finally, the mean Harris hip score improved from 39.6 preoperatively to 80.9; 22 of the 23 cages (including 1 re‐revision case) were considered stable and without migration based on the radiographic data at the final follow‐up^18^.

### *Tumor*

The treatment of peri‐acetabular malignant bone tumors is a great challenge to orthopaedic surgeons. Historically, the common method for managing peri‐acetabular malignant bone tumors was hindquarter amputation or external hemipelvectomy^52^. However, the optimal reconstruction method remains controversial because of the relatively high rate of associated complications. Currently, with advances in radiotherapeutic, chemotherapeutic, and surgical techniques, limb‐salvage surgery has become an accepted treatment. In 2007, Dai *et al*. reported their experience in customized hemi‐pelvic prostheses implantation in 10 patients who underwent internal hemi‐pelvectomy for extensive pelvic tumors. Dai *et al*. 3D‐printed the pelvic models of the patients based on their CT images. After the simulated bone resection was done on the model, the hemi‐pelvic prosthesis was designed and manufactured. However, the prostheses in these reports were still manufactured through a mold‐melted founding process instead of using a 3D printing machine^53^. Liang *et al*. (2017) examined the feasibility of using 3D printing technology for patients with a pelvic tumor who underwent reconstruction^54^. Based on Enneking's classification of bone defects, Liang *et al*. used 3 kinds of 3D‐printed prostheses for patients with different classification. A total of 35 patients underwent resection of a pelvic tumor and reconstruction using 3D‐printed prostheses. Finally, the application of 3D printing technology facilitated the precise matching and osseointegration between implants and the host bone. The use of 3D‐printed pelvic prostheses for reconstruction of the bony defect after resection of a pelvic tumor did not take extra time or involve increased blood loss and provided good short‐term functional results without additional complications^54^.

### *Osteonecrosis of The Femoral Head*

Total hip arthroplasty is not recommended for early osteonecrosis of the femoral head (ONFH), especially in young patients. Therefore, hip preservation becomes an important therapeutic principle. Zhang *et al*. applied a new 3D‐printed titanium metal trabecular bone implant to replace the necrotic bone of patients with early osteonecrosis of the femoral head. This kind of 3D‐printed titanium metal trabecular bone has high volume porosity (>80%) and allows complete communication among the pores. Thirty patients who underwent surgery for ONFH were selected in Zhang's study. First, surgeons removed the bone of the greater trochanter with a circular bone removal apparatus and gradually reamed the hole. The same diameter was selected for the final 3D‐printed titanium metal trabecular bone implant. Then they fill the pores of the 3D‐printed titanium metal trabecular bone with autologous cancellous bone harvested from the greater trochanter or bone allograft harvested in the area of the femoral head necrosis in the femoral head. Finally, they placed the 3D‐printed titanium metal trabecular bone in the right position after determining the insertion depth. The results show that 3D‐printed titanium metal trabecular bone cannot completely stop the progress of ONFH but may be effective in delaying its progression^14^.

## Patient‐Specific Instrument

Patient‐specific instruments offer an alternative to computer navigation for intraoperative assistance. Allied with the preoperative CT, the shape of PSI allowed unique positioning on the surgical accessible bone surface as determined by the surgeons utilizing the CAD software to make accurate bone cuts during surgery^47^. To improve the accuracy of the PSI, several aspects should be considered, including CT image resolution, suitability of anatomical land marking, and manufacturing errors. Recently, an experimental *in vitro* study including 60 cases by Lee *et al*. showed that a CT‐based navigation system with PSI was more accurate and consistent than the conventional technique for assessment of femoral component position^55^.

### *Fracture*

**Figure 4:**
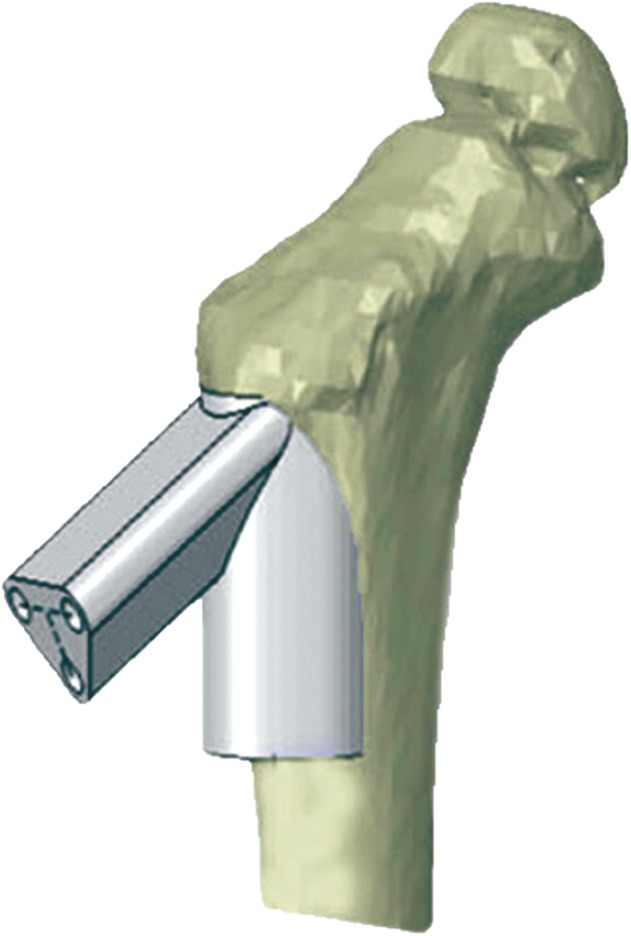
After reduction of the femoral neck fracture through software simulation, a navigation instrument was designed by the computer to conform to the proximal femur. The instrument provided optimal screw path and screw length for guiding pins and screws, which could be used to fix the Locking Compression Pediatric Hip Plate onto the fractured femoral neck during surgery^56^.

Zheng *et al*. created a drill template based on CAD and 3D printing technology for the placement of screws in a locking compression pediatric hip plate (LCP‐PHP) (Fig. 4). Using the CT data, the proximal femur model was created by a 3D printer. Fracture reduction and the placement of the screw in the femoral neck and the LCP‐PHP were simulated by the computer. Then a navigation template was designed by the software to conform with the proximal femur. The guide pins and the screws were inserted with the aid of the navigation template in the operation. This technology can reduce intraoperative damage to the femoral neck epiphysis, decrease operation time, reduce intraoperative hemorrhage, and decrease patients’ radiation exposure during surgery^56^. Furthermore, Maini *et al*. developed a control study to evaluate the accuracy of virtual surgical planning for a patient‐specific pre‐contoured plate in acetabular fracture fixation. In the PSI group (12 patients), CT‐based virtual surgical planning was done using MIMICS software to form virtually pre‐contoured plates, which were 3D‐printed to act as templates over which 3.5‐mm reconstruction plates were manually contoured preoperatively and used for fixation. The results indicated that the duration of surgery was shorter and total blood loss was less, and anatomical reduction rate in the PSI group was higher than that in the conventional group^13^.

### *Development Dysplasia of the Hip*

Zheng *et al*. explored the feasibility of a 3D‐printed navigation template in proximal femoral varus rotation and shortening osteotomy for older children with DDH in a control study. Navigation templates were designed and used for 12 DDH patients, while another 13 patients underwent the same surgery but without the navigation template. According to the preoperative measurements and in comparison with the contralateral parameters, the femoral varus angle, the rotation angle, and the length of bone to be cut were determined to devise the 3D‐printed navigation template. The results showed that the template‐guided group achieved a better outcome: operation time (21.08 min *vs* 46.92 min), number of X‐ray exposures (3.92 *vs* 6.69), and occurrence of femoral epiphysis damage (0 *vs* 0.92) were significantly decreased (*P* < 0.05)^57^.

### *Tumor*

**Figure 5:**
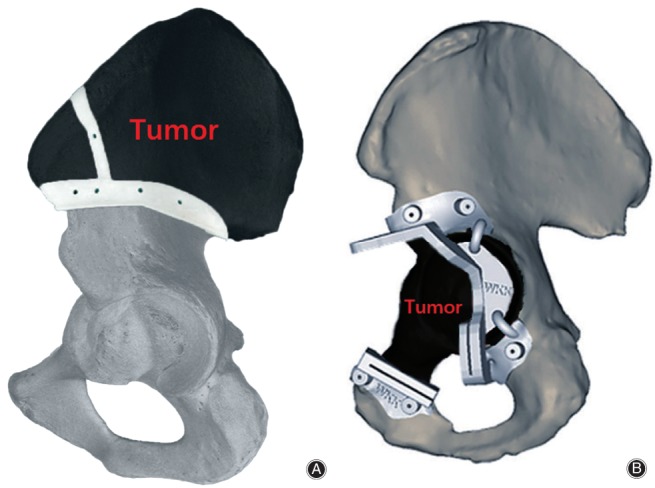
A, A 3D‐printed patient‐specific instrument (PSI) has a cutting slit that matches the planned resection planes. The black region represents the tumor. The K‐wire holes can stabilize PSI to the bone^61^; B, A 3D‐printed PSI, which can be used as an osteotomy guide plate. The black region represents the tumor. The flanges of the PSI allow a unique position on the bone surface; the K‐wire holes on the flanges can stabilize PSI on the bone 47.

In 1987, Jansen *et al*. used saddle prostheses to treat 17 patients with peri‐acetabular tumors. After a mean of 12.1 years of long‐term follow‐up, they reported a mean MSTS‐93 score of 47% and an 82% complication rate. Therefore, they deemed the saddle prostheses unsuitable for reconstruction following periacetabular tumor resection^58^. Therefore, prosthetic design and prosthetic reconstruction after tumor resection warrant study. Sallent *et al*. carried out a cadaveric study to assess the accuracy of PSI‐guided osteotomies compared to a standard manual technique in pelvic tumor resection. Sallent showed that computer‐assisted planning and PSI improved accuracy in pelvic tumor resections, bringing osteotomy results closer to the parameters set in preoperative planning, as compared with standard manual techniques^59^. In a case study, a 3D model of a pelvis with a large chondrosarcoma allowed the production of custom osteotomy guides, which aided tumor resection with adequate margins^60^. In 2015, Wong *et al*. described a comprehensive workflow of performing a partial acetabular resection in a patient with pelvic chondrosarcoma and reconstruction with a custom pelvic implant in a one‐step operation. A CAD custom implant was prefabricated with 3D printing technology and was biomechanically evaluated soon afterwards. Then a multi‐planar bone resection was virtually planned preoperatively. The 3D‐printed PSI were used to reproduce the same planned resection (Fig. 5A). In the end, the histology of the tumor specimen showed a clear resection margin. The errors of the achieved resection and implant position were deviating (1–4 mm) from the planning^61^. In a retrospective study, Wang *et al*. treated 11 patients with peri‐acetabular malignant bone tumors using PSI during en bloc resection (Fig. 5B). No local tumor recurrence was observed in these patients^47^. Holzapfel *et al*. applied both customized prostheses and PSI to treat 56 patients with periacetabular tumors. Preoperatively, a 3D model of the patient's pelvis was fabricated according to data obtained by high‐resolution CT. Based on the marked resection planes, special prosthesis and osteotomy guides were designed. Ten of 56 patients (17.9%) experienced local recurrence after a mean of 8.9 months. The surgical approach assessed in Holzapfel's study simplifies the process of tumor resection and leads to acceptable clinical and oncological outcomes^62^.

### *Osteonecrosis of The Femoral Head*

The conventional method of core decompression combined with porous bio‐ceramics rod is usually performed under C‐arm fluoroscopy for the treatment of early osteonecrosis of the femoral head. However, Wang *et al*. introduced a new method that uses 3D printing and patient‐specific instrument to enhance drilling accuracy during core decompression surgeries in a cadaveric study. They used 12 cadaveric human femurs to simulate early‐stage ischemic necrosis. Three positioning Kirschner wires were drilled into the top and bottom of the greater trochanter. A 5‐mm glass ball was placed in the femoral head as the target spot for decompression. The specimen was then subjected to CT and imported into the MIMICS software to construct a 3D model including the target. The best core decompression channel was then designed on the 3D model. A navigational template for the specimen was designed to guide the drilling channel. The specimen‐specific navigation template was installed on the specimen using positioning Kirschner wires. Drilling was performed using a guide needle through the guiding hole on the templates. The distance between the end point of the guide needle and the target was measured to validate the patient‐specific surgical accuracy. Results showed that core decompression using a patient‐specific instrument is a reliable and accurate technique^63^. Ulteriorly, Li *et al*. devised a customized 3D printing guide plate, instead of C‐arm fluoroscopy, to guide the core decompression operations in patients. The 3D printing guide plate could be tightly attached to the proximal part of the femur during operation, and one Kirschner wire could be inserted into the pinhole on the guide plate to obtain a core decompression position. Then a bony channel up to about 5 mm depth underneath the articular cartilage surface was established by using a core reamer. Li *et al*. proved that 3D printing guide plate could shorten operation time and fluoroscopy time and decrease intraoperative blood loss^15^.

### *Fibrous Dysplasia*

In addition to the various diseases mentioned above, shepherd's crook deformity due to fibrous dysplasia may also influence hip joint sometimes. Wan *et al*. devised a patient specific osteotomy template to anchor with k‐wires onto the most suitable surface of femur, then osteotomy was made by using an electric saw along with the designed osteotomy line of the template. A total of 10 patients of shepherd's crook deformity were enrolled in Wan's study. The neck shaft angle was corrected from a mean value of 88.1° (range, 73°–105°) preoperatively to a mean value of 128.5° (range, 120°–135°) postoperatively, showing that 3D printing osteotomy templates could make a positive correction of shepherd's crook deformity.^64^

## Discussion

**Figure 6:**
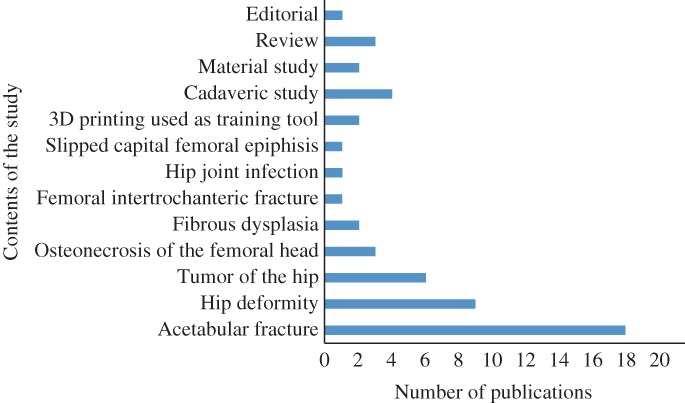
Numbers of published articles by content of the study.

**Figure 7:**
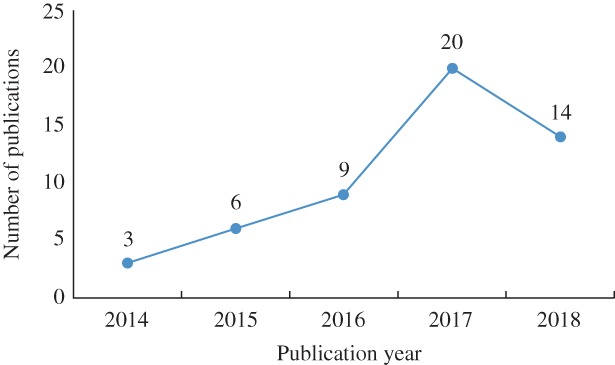
A graphic history of the published studies addressing both 3D printing and hip joint disease in the past 5 years.

Numbers of published articles by content of the study are shown in Fig. 6, and a graphic history of the published studies addressing both 3D printing and hip joint in the past 5 years is shown in Fig. 7. Manufacturing of 3D models has many advantages. First, 3D‐printed pelvic models provide a multi‐angle view, making it easier for surgeons to understand the preoperative situation and to further classify fractures in cases of complex fractures^65^. Second, 3D‐printed models can be used for preoperative simulation. They are useful in choosing the best approach, pre‐bending the appropriate length plates, determining the optimal position of plates, selecting the most effective angle and length of screws, and deciding on the most prudent preoperative plan. In fact, 3D printing techniques could improve surgeons’ efficiency in the operating room, shorten operative times, and reduce exposure to radiation^26^. Another advantage of 3D models is their use as learning tools for educating students and young surgeons^66^, ^67^. 3D‐printed models can reproduce assorted patterns of fractures for novices to practice complex surgeries.

As for custom prostheses, 3D printing technology can be used to fabricate a metal surface with a porous scaffold. The porous scaffold allows the host bone to grow inside the construct to achieve a stable biomechanical reconstruction^68^. In addition, non‐custom implants can lead to serious complications caused by significant bone resorption secondary to stress shielding; however, Arabnejad *et al*. designed a fully porous 3D‐printed titanium implant, which can reduce the amount of bone loss secondary to stress shielding by 75% compared to a fully solid implant^69^. Another material study conducted by Kim *et al*. showed that the use of a 3D‐printed polymeric implant could act as a controlled drug delivery vehicle by using built‐in reservoirs and a network of micro‐channels as well as by incorporating antibiotics directly into the polymer during the manufacturing stage^70^. Without limitation of the shape of traditional prostheses, the shape of 3D‐printed prostheses can adjust to different kinds of diseases^71^. Having excellent design capability, 3D‐printed prostheses can solve the situation when it is difficult to lay and fix several traditional prostheses together^10^.

Patient‐specific instruments can create a rapid and accurate osteotomy without the aid of computer navigation, which facilitates precise matching of the implant to the section and decreases operative time, invasiveness, and intraoperative surgeon decision‐making^47^. The use of 3D printing model for surgical planning, engineering software for implant design and validation, together with 3D printing technology for implant and PSI fabrication makes it possible to develop a personalized, biomechanically evaluated implant for accurate reconstruction after a pelvic tumor resection in a one‐step surgery^61^.

However, 3D printing also has limitations, such as its resolution (0.1 mm at the most) and the difficulty of including cartilage and soft tissue, which are excluded in the process of segmentation of the radiological image^9^. 3D models cannot locate important blood vessels and nerves accurately, and even minimal deviation can affect outcomes intraoperatively because of the differences in blood vessels, nerves, and the musculoskeletal system^28^. Therefore, CT angiography is still vital for determining the position of important arteries accurately, such as the superior gluteal artery and the corona mortis. Moreover, the procedure of 3D printing can be influenced by artificial factors. If the key fragment were absent because of the carelessness of the radiologist, the intraoperative reduction and fixation might be different to what is expected based on the preoperative plan, thus affecting the surgeon's judgment during the surgery. The cost of 3D printing is relatively expensive. At present, the cost for a 3D model of the hip and hemipelvis can vary from $200 to $1000 depending upon factors such as the materials used, the size of the print, and the type of printer used^17^. However, some authors maintain a positive view; they believe that we no longer need to depend on intermediaries for using 3D printing technology. Excluding the investment in the printer, the costs per preplanned model are relatively cheap^28^. In addition, in the manufacture of individualized prostheses, the clinically useful material is limited to metal, ceramic, and plastic. Research on other materials such as collagen, chondroitin sulfate, hyaluronic acid, and hydroxyapatite is still in the laboratory stage. However, with the development of tissue engineering and digital medicine, new materials and technologies, we anticipate that 3D printing technology will be widely used in the field of joint surgery^72^.

Currently, most of the articles on 3D printing have several mutual shortcomings. First, there are just a few articles mentioning custom prostheses or PSI (14 in 53 articles), which limits the study. Second, the short follow‐up period of the study does not account for some biomechanical complications that may appear when we follow these patients for a longer time. Third, a great many articles are constricted by their retrospective design and the lack of a control group (only 5 in 53 articles are control studies). Besides, several studies have reported that use of PSI did not show an improvement in overall limb alignment or shortened operative time compared with conventional instruments^73^. Therefore, further study is needed to assess the clinical efficacy of PSI. Research on 3D printing is still in its infancy. With further in‐depth study of 3D printing comes the potential for improved treatment of complex hip joint disease.
